# Adolescents and young adults’ concerns under stress, the COVID-19 pandemic: a Portuguese cross-sectional study

**DOI:** 10.3389/fpsyg.2023.1260070

**Published:** 2023-10-18

**Authors:** Carlos Franclim Silva, Daniel Beirão, Luísa Sá, Paulo Santos

**Affiliations:** ^1^MEDCIDS – Department of Community Medicine, Information and Health Decision Sciences, Faculty of Medicine, University of Porto, Porto, Portugal; ^2^CINTESIS@RISE, MEDCIDS, Faculty of Medicine, University of Porto, Porto, Portugal

**Keywords:** adolescent, young adult, COVID-19 pandemic, concerns, behavior, stress, health literacy

## Abstract

**Background:**

Adolescents and young adults are a critical target regarding reducing healthrisk behaviors’ adoption, particularly in a stressful context such as the COVID-19 pandemic. Surveying their perceptions and behavioral changes may lead to a revised health promotion approach.

**Objectives:**

This study aims to describe adolescents’ and young adults’ early reactions to a stressful event, the COVID-19 pandemic, characterizing their social determinants and preferences, such as sources of information, experienced symptoms, habits, and main concerns regarding themselves, their relatives, and the community. We intended to explore the association of their concerns and significant determinants such as age, gender, education, source of information, feelings of fear, prospecting the implications of communication, and individual and social determinants in managing stressful events.

**Methods:**

We conducted a cross-sectional, population-based, self-report survey of 3,898 individuals aged between 16 and 24  years from Portugal.

**Results:**

The main concerns about COVID-19 were the possibility of transmitting to someone and permanent social changes. Our findings present significant differences according to gender, education, age, and expressing fear.

**Conclusion:**

The pandemic deteriorated baseline community inequalities. Young people appreciate official information and are available to contribute to society’s safety. However, valuing official information is associated with deeper expressed concerns. Therefore, official information should include strategies to reach young people, promote healthier choices, and avoid distress and disinformation.

## Introduction

Young people are prone to experimentation and exploration, leading to a high risk of adopting unhealthy behaviors (De-Sanctis et al., 2014), potentially impacting current and future health status. Preventive medicine can be considered and is suitable for all people, regardless of their age and condition. Nevertheless, people in a stage of life that comprehends extending their global skills and becoming responsible for their choices could be a good target. Health promotion, including education and improving literacy, is the key to effective change in behaviors and lifestyles (Santos et al., 2017). Health literacy competence is accessing and using information regarding healthy habits, good living conditions, and proper health services utilization (Nutbeam, 1998). Most adolescents’ and young adults’ health problems are preventable and should be the focus of assessment and intervention (Neinstein, 2014; Kann et al., 2016). Primary health care services are particularly well positioned to do it, as they are the first level of access to the system. However, family physicians tend to assign low priority to young people (Hetlevik et al., 2010), especially when the workload is high and the remaining medical care capacity is low.

Adolescents and young adults present brains underdeveloped regarding affect regulation, prefrontal cortex, and judgment and reasoning skills are fully developed at about 26 years (Bell and McBride, 2010). They are particularly susceptible to the social environment and adopt unhealthy routines. Additionally, they present more remarkable plasticity and are available for adaptive social learning (Luna et al., 2013; Somerville, 2013). Social support and environment are critical to young people’s emotional regulation and adoption behaviors. Many risky behaviors are frequently associated; they present a common underlying condition or cause-effect dynamics, enhancing the value of a holistic approach (Peterman et al., 2015). Therefore, studying young people’s expectations and habits during a stressful event may lead to information regarding that specific condition and the general impact of stress.

The COVID-19 infection was a slight disease for adolescents and young adults, leaving them behind in the health system’s priorities. Moreover, the measures imposed to contain the pandemic, like physical isolation and quarantine, significantly affected their physical, mental, and social dimensions (Usher et al., 2020). Psychological distress and other mental health problems were common in those forced to quarantine (Hawryluck et al., 2004; Liu et al., 2012; Taylor et al., 2020), expressed by symptoms of confusion, anger, frustration, boredom, fear of being infected or infecting others, stigma, and fear of lacking access to basic supplies or financial losses (Brooks et al., 2020; Diotaiuti et al., 2021a). Emotions are essential drivers in decision-making (Lerner et al., 2015), embracing behavioral, physiological, and biochemical mechanisms (Damasio and Carvalho, 2013). Fear is an emotion attached to the action to preserve integrity when facing a perceived threat (Velasco et al., 2019), embracing both adaptive and maladaptive responses (Gunduz-Cinar, 2021). This emotion is critical in youth reaction to a global threat such as the COVID-19 pandemic (Giordani et al., 2022). During the COVID-19 lockdown, perceived self-efficacy and distress were associated with predominant cognitive appraisal; those oriented toward learning and solidarity appeared to impact positively, in opposition to fear-oriented (Diotaiuti et al., 2023). There was a dominant fear of death, suffering, and inability for the individuals and their loved ones, extended for too long without a clear and strong leadership of national authorities. Government communication impacts youth’s perception of risk and their ability to manage critical situations, such as the COVID-19 pandemic (Diotaiuti et al., 2021b). During the pandemic, maladaptive fear was common, leading to distress and anxiety and negatively impacting daily routines, such as eating behaviors and physical activity, which also affects mental health (Flores et al., 2018; Ferrara et al., 2022; Galli et al., 2022). The symptoms and emotions expressed during a stressful event in this life stage may impact behavior changes and long-term adverse consequences on mental health problems that may track into adulthood (Meherali et al., 2021).

Youngers are particularly vulnerable to stress factors that affect the construction of socialization. It is crucial to understand the adaptive and maladaptive responses during a stressful event, such as the COVID-19 pandemic, and their relationship with habits, perceptions, and health preferences to adapt facilities to the real needs of younger and provide adjusted answers. Adolescents and young adults’ concerns about the COVID-19 pandemic are among their priorities and efforts to face the perceived threat (positive response) or, on the other hand, fear and distress affecting their well-being (negative answer). Describing these concerns and their related factors allows us to identify individuals at risk of adverse emotional reactions during and after the pandemic or likely to engage in safety behaviors during stressful events. We intended to study potential protective and threatening factors to express stress and feeling worried about, such as age, gender, education, the feeling of fear, and valuing information about COVID-19. Age, gender, and education are common explanations of outcome discrepancies, particularly in the stage of life where a significant global development process occurs. Feeling fear is a primary emotional expression attached to the action to preserve integrity when facing a perceived threat, such as the COVID-19 pandemic; it is a critical driver in decision-making, and it is associated with psychological stress. The official information includes authorities’ recommendations to manage the pandemic, such as social isolation, and data from the pandemic spreading and its mortality, a potential cause of stress (Usher et al., 2020). These findings may enhance community and health services responses, and communication, enhancing health literacy strategies.

## Aims

This study aims to describe adolescents’ and young adults’ early reactions to a stressful event, the COVID-19 pandemic, characterizing their social determinants and preferences, such as sources of information, experienced symptoms, habits, and main concerns regarding themselves, their relatives, and the community. We intended to explore the association of their concerns and significant determinants such as age, gender, education, source of information, feelings of fear, prospecting the implications of communication, and individual and social determinants in managing stressful events. Therefore, we aimed to explore the target population’s appraisal of this stressful event and authorities’ communication strategies to face it, prospecting critical determinants to enhance health literacy and health prevention policies.

## Methods

We conducted a cross-sectional study, with analytic intention, on Portuguese youngers between 16 and 24 years old through an online survey distributed from April/2020 to July/2020.

### Sampling

Portuguese National official data shows Portugal had 991,194 inhabitants aged 16 to 24 in 2019. We used stratified sampling, maintaining the proportion of main Portuguese regions according to the classification of territorial units for statistics (NUTS II). Assuming an unknown distribution, we estimated a sample size of 2,396 participants for a 95% confidence interval and a maximum error of 2%. Table 1 shows the distribution of the sample according to the main regions.

**Table 1:** Sample distribution by Portuguese main regions.

Region	Population	Expected sample
North	359,695	869
Center	207,669	502
Lisbon and Tagus valley	262,550	635
Alentejo	63,637	154
Algarve	40,996	99
Azores	27,980	68
Madeira	28,667	69
Total	991,194	2,396

### Participants

All adolescents and young adults aged 16 to 24 living in Portugal were eligible for participation. We excluded those who did not speak or understand the Portuguese language and those without the physical or mental ability to fill out the questionnaire.

The total sample included 3,898 participants, largely above the calculated sample size due to the attempt to reach the sample size in the cluster of the regions of Azores and Madeira. The mean age was 19.1 years old (Table 2). The majority were female (71.2%), had Portuguese nationality (94.8%), were students (87.2%), and frequented or concluded their secondary education (65.3%).

**Table 2:** Demographic characteristics.

Age	Years, mean ± SD	19.1 ± 2.4
Gender	Female (n)	2,777
	Male (n)	1,112
	Gender-diverse people (n)	9
Education (Last school year attended)	Primary (n)	326
	Secondary (n)	2,373
	Higher (n)	935
Labor status	Student (n)	3,399
	Worker (n)	259
	First-time jobseeker (n)	80
	Unemployed (n)	75
	Student worker (n)	64
	Other (n)	19
Nationality	Portuguese (n)	3,688
	Other (n)	204
Residence	North (n)	1,259
(Region)	Center (n)	559
	Lisbon and Tagus Valley (n)	1,553
	Alentejo (n)	218
	Algarve (n)	236
	Azores (n)	17
	Madeira (n)	29

### Procedures

The ethics committee of the Faculty of Medicine of the University of Porto and Hospital of São João approved this study (process number 123/2020). The current study complied with the Declaration of Helsinki and the Oviedo Convention and the ethical principles of the American Psychological Association on the rights of individuals participating in biomedical investigations. We followed the non-discrimination principles of the Charter of Fundamental Rights from the European Union Agency for Fundamental Rights.

Using a convenience sampling approach, participants were recruited from the community and via social media platforms. We sent the invitations through the mailing list of academic, sports, artistic, governmental, social, leisure, and professional institutions and widely used social network advertisements. We directly contacted 2,268 institutions to spread the invitation by e-mail and phone call at least three times, including the Portuguese Youth Card organization, which has about 40,000 affiliated adolescents and young adults spread nationwide. We contacted institutions presenting adolescents and young adults as the target population, formally recognized by official authorities, the Ministry of Labour and Social Solidarity, the Ministry of Science, Technology and Further Education, Ministry of Education, including the Youth and Sports Department, of the Portuguese Government, accessing official online open access information, and searching institutions contacts on online generic free search engines and social networks. Participants have not received any compensation for participating in the questionnaire. We explain the authors’ affiliations, the main topics, and the research aims before participants fill out the questionnaire.

The self-administered online survey was available between April/2020 and July/2020.

### Questionnaire

The questionnaire embraced four sections: the first one about the COVID-19 pandemic, displaying three questions about main worries and primary sources of information; the second one exploring habits, occupational and leisure patterns, through two questions; the third section presented five questions about health and recent symptoms; and last section asking for generic sociodemographic data, through 6 questions. We opt for a clear and easy speech, using sentences like “What worries you most about the COVID-19 pandemic?.” We evaluated adolescents and young people’s main concerns about the COVID-19 pandemic, reported in the week before: fear of being infected with COVID-19 and transmitting it to someone else, fear of losing freedom, the chance of developing a mental health problem or other health conditions, lack of access to food, accommodation, healthcare services, leisure and culture, academic and labor activities and spending time with family and friends, feelings of insecurity, violence and economic insecurity, and permanent social changes. We select variables according to the previous literature’s main findings (Flores et al., 2018; Brooks et al., 2020). We evaluated the perception of worry about each item through a 5-point Likert scale, raged from 1, “I worry a little about it,” to 5, “I worry a lot about it,” and dichotomized as a positive case of worry (4 or 5 points) or negative (all others).

We asked for symptoms commonly associated with anxiety (16) and mood disorders (17), including headache, back pain, abdominal pain, dizziness, tiredness, sadness, sleep disturbance, nervousness, irritability, and feeling the primary basic emotion of fear, considering a positive case if the participant had felt it at least once in the past week. The perceived changes in behavioral patterns included questions regarding alcohol, tobacco, and other psychoactive substance consumption. We studied the habits of psychoactive substance consumption as possible markers of maladaptive responses to stress through a 7-point Likert scale, which ranged from −3, “it got a lot worse,” 0, “equal,” to +3, “much better.”

General characterization included gender, age, nationality, current residence county, graduation, and labor status. We use Heidari recommended definitions regarding the sex and gender approach (Heidari et al., 2016).

To check the questionnaire’s validity and comprehensibility, we performed a pilot test on a group of volunteers aged 25–30 who were not part of the study population.

### Data analysis

We used Kolmogorov–Smirnov test to evaluate the Normal distribution. Using the modified Wald method, we calculate prevalence and 95% confidence intervals (CIs). The inferential analysis used Student’s *t*-tests, nonparametric tests, and Chi-square or Fisher test, depending on the variables. Multivariate analysis used a binary logistic regression model to estimate the relationship between factors and dependent variables. The significance level was set at 0.05. Data were encoded and registered in a Microsoft Office Excel 2010 database and analyzed using IBM SPSS Statistics, version 28.0 (IBM Corp., Armonk, NY, USA).

## Results

The main concerns about COVID-19 were the possibility of transmitting COVID-19 to someone, important to 93% of the participants; permanent social changes, valorised by 73%; access to healthcare facilities by 72%; access to academic activities by 70%; and access to time with family and friends by 66%. Tables 3, 4 show the main outcomes of habits and concerns during the COVID-19 pandemic. General Directorate of Health (GDH) online information was the most valuated source (88%), followed by TV (70%).

**Table 3:** COVID-19 pandemics habits and concerns.

	*n*	Proportion (%)*	95% CI
Main sources of information			
Television	2,609	70	69–71
Radio	1,478	41	39–43
Social network	1,527	39	38–41
GDH online information	3,356	88	87–89
Internet search engines	1,584	42	40–44
Books, journals, magazines	1,616	44	42–46
Main concerns			
Being infected with COVID-19	2,265	59	57–61
Transmitting COVID-19 to someone	3,551	93	92–94
Developing a mental health problem	2,077	56	54–58
Developing other health problems	1,909	52	50–54
Healthcare services access	2,710	72	71–73
Food access	1,369	36	35–38
Accommodation access	862	23	22–24
Insecurity	1,520	41	39–43
Violence	974	25	24–26
Family time or friends’ access	2,510	66	64–67
Economic problems	2,200	57	55–59
Leisure and cultural activities access	1,627	43	41–45
Academic activities access	2,610	70	69–71
Laboral activities access	1,866	56	54–58
Freedom	2,234	59	57–61
Permanent social changes	2,748	73	72–74
Symptoms (past week)			
Tiredness	2,201	57	55–59
Sadness	2,192	57	55–59
Sleep disturbs	1,800	47	45–49
Feeling nervous	2,242	59	57–61
Feeling Irritable	2,142	56	54–58
Fear	1,065	29	28–30

n, number of positive cases; CI, confidence intervals; *adjusted to residence region.

**Table 4:** Psychoactive substances consume patterns.

Psychoactive substance	Consume pattern	*n*	Proportion (%)*	95% CI
Alcohol	Increased	3,465	23	22–24
	Remained	3,397	65	63–67
	Diminished	3,283	12	11–13
Tobacco	Increased	3,127	15	14–16
	Remained	3,190	77	76–78
	Diminished	3,225	8	07–09
Other psychoactive substance	Increased	3,042	12	11–13
	Remained	3,119	84	83–85
	Diminished	3,425	4	03–05
Any psychoactive substance	Increased	3,556	27	26–28
	Remained	3,011	63	61–65
	Diminished	2,533	3	02–04

n, number of positive cases; CI, confidence intervals; *adjusted to residence region.

In the past week, most participants expressed tiredness, sadness, nervousness, or irritability. Feelings of fear were identified in 29% of the total, more in females than in males (*p* < 0,001). Psychoactive substance consumption increased during the pandemic in 27% of participants, primarily due to alcohol-containing beverages.

Table 5 shows the impact of age, gender, higher education attendance, valuing GDH online information, and expression of fear in the past week in the worry about COVID-19. Age was significantly related to most concern about economic problems and access to work activities, and less to access to food. Older presented higher levels of psychoactive substance consumption. Females were more concerned about being infected or transmitting COVID-19, developing a health problem, accessing food and healthcare services, or academic and work activities. They also presented more worry about insecurity, violence, economic issues, and permanent social changes. Higher education was related to fewer concerns about being infected with COVID-19, accessing health care services, accommodation, food, insecurity, and violence. Participants who valued online information from the General Directorate of Health presented significantly deeper concerns regarding all topics except leisure and cultural activities. Additionally, valuing GDH was associated with lower odds of worsening psychoactive substance consumption patterns. Expressing fear in the past week was related to concerns about all topics.

**Table 5:** Logistic regression model adjusted to age, gender, education, valuing GDH and fear.

	Main concerns															
	Being infected with COVID-19	Transmitting COVID-19 to someone	Developing a mental health problem	Developing other health problems	Healthcare services access	Food access	Accommodation access	Insecurity	Violence	Family time or friends’ access	Economic problems	Leisure and cultural activities access	Academic activities access	Laboral activities access	Freedom	Permanent social changes
	OR* (95% CI)	OR* (95% CI)	OR* (95% CI)	OR* (95% CI)	OR* (95% CI)	OR* (95% CI)	OR* (95% CI)	OR* (95% CI)	OR* (95% CI)	OR* (95% CI)	OR* (95% CI)	OR* (95% CI)	OR* (95% CI)	OR* (95% CI)	OR* (95% CI)	OR* (95% CI)
Age (18–24)	–	–	–	–	–	0.79 (0.67–0.93)	–	–	–	–	1.29 (1.11–1.49)	–	–	1.56 (1.34–1.83)	–	–
Gender (female)	1.63 (1.40–1.91)	1.53 (1.17–2.00)	1.65 (1.41–1.93)	1.32 (1.14–1.55)	1.80 (1.53–2.12)	1.19 (1.02–1.41)	–	1.53 (1.29–1.81)	1.54 (1.28–1.86)	–	1.47 (1.26–1.71)	–	1.69 (1.44–1.98)	1.36 (1.15–1.60)	–	1.59 (1.35–1.87)
Higher education	0.77 (0.66–0.90)	–	–	–	0.84 (0.70–1.00)	0.57 (0.48–0.69)	0.66 (0.55–0.81)	0.81 (0.68–0.95)	0.78 (0.65–0.94)	–	–	–	–	–	–	
Valuing GDH	1.60 (1.30–1.98)	3.46 (2.59–4.64)	1.59 (1.28–1.98)	1.60 (1.28–1.99)	2.04 (1.64–2.54)	1.40 (1.12–1.77)	1.32 (1.02–1.72)	1.37 (1.08–1.73)	1.49 (1.14–1.94)	1.55 (1.25–1.92)	1.61 (1.30–1.99)	–	1.74 (1.40–2.17)	1.70 (1.36–2.14)	1.52 (1.23–1.87)	1.49 (1.19–1.86)
Fear	1.49 (1.27–1.75)	1.52 (1.10–2.10)	2.78 (2.36–3.28)	1.91 (1.64–2.24)	1.57 (1.31–1.89)	1.41 (1.21–1.65)	1.31 (1.11–1.56)	3.33 (2.85–3.91)	1.56 (1.32–1.84)	1.96 (1.65–2.32)	1.50 (1.28–1.75)	1.29 (1.11–1.50)	1.80 (1.50–2.16)	1.66 (1.40–1.96)	1.74 (1.49–2.04)	1.75 (1.45–2.10)

OR, Odds Ratio; CI, Confidence Interval; GDH, General Directorate of Health; *p < 0.05.

## Discussion

Adolescents and young adults mostly expressed concerns about transmitting COVID-19 to someone else, the risk of permanent social changes, the access to healthcare services and academic activities, and living together with family or friends. Psychological symptoms were frequent, with high levels of fear and substance consumption. These findings corroborate the significant impact of the COVID-19 pandemic on youth well-being and depend on the socio-demographic factors, the influence of the General Health Directorate, the main source of information, and the expression of fear emotion.

COVID-19 affects mainly elders and patients with comorbidities. The disease is rare in adolescents and young adults, explaining why the concern about transmitting the infection to others is more frequent than being infected. This is in line with Leão et al. (2021) a Portuguese study during the first month of the pandemic in Portugal stated that transmitting COVID-19 to someone is a significant concern, as our results indicate. In Europe, Sabat et al. (2020) suggested that the epidemic was a stressor to health and economic topics, with substantial differences between regions and age groups. Portugal showed a higher prevalence of worries concerning the health system, losing someone, recession, and small business, above 80%, and between 60 and 80% of increased concerns about food access, unemployment, and an egoistic society. The school closure worry prevalence was under 40% in the evaluated countries, globally lower than in the present study, probably due to population age discrepancies or distinct education institutions’ strategies to adapt to different teaching conditions. Along the same line, despite the different age groups, healthcare services and economic problems are also prevalent concerns in our study.

The COVID-19 pandemic was a significant public health problem that reached the economy, community, media approach, environment, ethics, and politics. Managing the COVID-19 pandemic implied promoting policies restricting individual freedom to face the spread of the disease, social distance to peers and family, occupational adjustments, and access to leisure activities, which is potentially stressful. Schou-Bredal et al. (2021) in Norway, found the preoccupation with family and friends as a significant population worry, raising concerns about the pandemic’s future and eventually permanent impact. Elmer showed the same in Switzerland, where this worry about family and friends was associated with higher depression (Elmer et al., 2020).

Access to healthcare facilities is a common concern for adolescents and young adults, consistent with other studies (Asmundson and Taylor, 2020). The constant news about overloaded health services, particularly in primary care (Lauriola et al., 2021), and the burnout of providers (Raudenská et al., 2020) may have contributed significantly to it, as the fear of getting the infection in the healthcare facilities (Soares et al., 2021). Additionally, the priorities established by the governments did not meet the expectations of both patients and providers, as in the case of abortion procedure access during the COVID-19 pandemic in the United States (Bayefsky et al., 2020). They contributed to increasing the distance between both and the worry in the population.

Most participants were students with significant constraints in academic activities, raising genuine concerns about access to school and academic achievement, where the impediments to peer relationships were also relevant. As expected, worries about jobs and economics were mainly present among the older. Preoccupation with access to food and other primary needs was lower than predicted by the relevance we could infer from Maslow’s hierarchy of human needs (van Lenthe et al., 2015). Nevertheless, attending higher education was associated with less stress regarding basic needs and security, fewer worries about being infected, and access to healthcare services, making education a social lift, even during the pandemic.

As in other studies, we found significant differences between gender, with females expressing more concerns about most of the evaluated items and more fear (Velasco et al., 2019; Schou-Bredal et al., 2021). These results confirm the gender differences in managing and facing potential threats, consolidating intrinsic abilities, and general behaviors. Worryingly, this may support some findings about the escalation of gender inequality (Brzezinski, 2021); with domestic violence, more social expectations with family caring responsibilities, and more vulnerability to economic consequences (Wenham et al., 2020; Ruiz-Pérez and Pastor-Moreno, 2021), females become more prone to negative mental health consequences (Elmer et al., 2020), with more suffering in a stressful situation like a pandemic. We asked for gender dimension on the used questionnaire, and not for sex dimension, attending to our main aims centered on behaviors and expectations.

In our study, official authorities’ information was a significant source of information. Social media was a minor one, like the findings in the European survey from Sabat et al. (2020). In Portugal, the official online information driven by the General-Directorate of Health was the main source regarding the COVID-19 pandemic for adolescents and young adults. The authorities transmission of information was adequate, and youth people got it and valued it, however health literacy is more than accessing valid knowledge, it embraces competences to understanding and using it (Silva and Santos, 2021). We find that official information was associated with higher concerns, probably distress, and not enhanced health literacy. The management of information and communication is crucial. It may stimulate an adaptive attitude facing a critical situation. Still, it can also make people avoid accessing information (Siebenhaar et al., 2020), especially if they find it to be unclear and confusing (Soares et al., 2021; Sun et al., 2022), out of step with people’s real needs, or undervaluing their concerns, ideas, and fears (Santos et al., 2015). The official information did not impact concerns about leisure and access to cultural activities concerning the less priority given to these aspects during the pandemic.

We found increased alcohol and drug consumption during a pandemic, similar to Horigian et al. (2021) in the United States. However, Alladio, in Italy, remembers that it is more an escalation of previous consumptions than an actual new behavior (Alladio et al., 2021). We evaluated the generic perception regarding patterns of consumption habits, and we did not describe the frequency, the quantities, or the usual context of consumption routines before and during the pandemics; that is a limitation.

We did not measure anxiety and stress through the usual available scales due to our general, broad, holistic, and preventive intention and aims. An extended questionnaire could induce participants to fail to respond to the whole questionnaire. However, it represents a significant limitation in analyzing the meaning of the perceived concerns and prospecting their adaptive or maladaptive value. Furtherer investigation is necessary to disclose it.

In our study, higher education is protective against concerns, although the fear and the information provided by national authorities may worsen them. We did not explore participants’ criteria to classify a source of information as important, its’ usefulness, considering protective strategies empowerment, facilitating access to main services such as health, or spreading official norms to behave and avoid problems with authorities, its’ credibility, and its suitability. This is a limitation targeting the health literacy approach.

The COVID-19 pandemic is a critical issue for youth, associated with concerns about transmitting COVID-19 to someone, permanent social changes, access to healthcare facilities and academic activities, and spending time with family and friends. Our findings present significant differences according to gender, education, age, and expressing fear. Females are more vulnerable to stress, and education is a protective factor. The pandemic deteriorates baseline services and community inequalities or inefficiencies. Adolescents and young adults appreciate official governmental information and are available to contribute to society’s safety. However, valuing official information is associated with deeper expressed concerns. Therefore, official information should include strategies to reach young people, promote healthier choices and avoid distress and disinformation.

The sampling method is a powerful strength of this study. This research introduces a novel approach to the youth perspective and concerns regarding the COVID-19 pandemic. We did not find similar studies focused on adolescents and young adults with such a broad scope. However, it highlights and explores the early attitude of facing a significant community health problem that may display their general attitude to feel and face the stress, vulnerabilities, and availabilities to contribute to personal and society’s security and well-being.

### Implications and contribution

The COVID-19 pandemic is a critical concern for youth, and it worsens baseline services and community inequalities. Young people appreciate official governmental information and are available to support community safety. Nevertheless, valuing official information is associated with more profound concerns. Official information should promote health literacy, healthier choices, facilitate proper access to health services, and avoid distress and disinformation. The COVID-19 pandemic is a stressful event, considering the morbidity of the disease and the implications of adopted strategies to face it. These findings may highlight young people’s perspectives and attitudes to facing a significant community health challenge, displaying their availability to contribute to the solution and their vulnerability regarding adopted strategies, including official communication. Additionally, people presenting sociodemographic vulnerabilities should be prioritized in preventive strategies.

### Limitations and future directions

We recruit participants using convenience sampling, however, through a robust method, strengthening the study’s applicability to a representative Portuguese Youth Population. We did not measure anxiety and stress through the usual available scales due to our generic broad aims. A prolonged questionnaire, embracing usual scales, could trigger participants to fail to complete the whole questionnaire. However, dismissing scales still represents a limitation. We did not used a valid questionnaire, nevertheless we conducted a pilot test to improve its comprehensibility. We did not explore participants’ appraisal of authorities’ information considering specific communication attributes; it could disclose critical communicational factors. Future studies may supply present limitations, upgrading fear and stress experience characterization and exploring youth perspective regarding authorities’ policies and communication.

## Conclusion

Youth is available to learn and solidarity regarding critical community health problems, such as the COVID-19 pandemic, and should be protected from potential distress by authorities’ management and communication strategies. Further works targeting the impact of authorities’ positive communication and measures procedures on youth distress manifestations and their commitment to promoting community safety is necessary. Additionally, we should describe experiencing fear emotion and the youth’s insight into fear’s impact and explore the youth’s perspective regarding authorities’ communication and their practical implications for health literacy.

## Data availability statement

The raw data supporting the conclusions of this article will be made available by the authors, without undue reservation.

## Ethics statement

The studies involving humans were approved by Comissão de Ética para a Saúde – Centro Hospitalar de São João, Faculdade de Medicina da Universidade do Porto (comissao.etica@chsj.min-saude.pt). The studies were conducted in accordance with the local legislation and institutional requirements. Written informed consent for participation in this study was provided by the participants’ legal guardians/next of kin. Written informed consent was obtained from the individual(s), and minor(s)’ legal guardian/next of kin, for the publication of any potentially identifiable images or data included in this article.

## Author contributions

CS: Conceptualization, Data curation, Formal analysis, Investigation, Methodology, Project administration, Resources, Software, Validation, Writing – original draft, Writing – review & editing. DB: Conceptualization, Formal analysis, Investigation, Software, Writing – review & editing. LS: Conceptualization, Methodology, Resources, Software, Writing – review & editing. PS: Conceptualization, Data curation, Formal analysis, Funding acquisition, Investigation, Methodology, Project administration, Resources, Software, Supervision, Validation, Visualization, Writing – original draft, Writing – review & editing.
